# Prevalence of perioperative asymptomatic venous thromboses of the lower extremity in 30 consecutive patients undergoing transsphenoidal surgery for Cushing’s disease

**DOI:** 10.1038/s41598-023-30070-8

**Published:** 2023-02-24

**Authors:** Torge Huckhagel, Gülsen Atlihan, Florian Langer, Jörg Flitsch, Roman Rotermund

**Affiliations:** 1 Department of Diagnostic and Interventional Neuroradiology, University Medical Center Göttingen, Robert-Koch-Straße 40, 37075 Göttingen, Germany; 2 Department of Neurosurgery, Division of Pituitary Surgery, University Medical Center Hamburg-Eppendorf, Hamburg, Germany; 3 Department of Vascular Medicine, University Medical Center Hamburg-Eppendorf, Hamburg, Germany; 4 Department of Hematology and Oncology, University Medical Center Hamburg-Eppendorf, Hamburg, Germany

**Keywords:** Endocrine cancer, Pituitary diseases, Epidemiology, Thromboembolism

## Abstract

Cushing´s disease is a rare endocrinological disorder which is caused by an adrenocorticotropic hormone secreting pituitary adenoma. The condition is associated with an increased risk for venous thromboembolism. While there exist reports on symptomatic venous thromboses complicating Cushing’s disease, the prevalence of incidental leg vein thromboses accompanying pituitary surgery for Cushing’s disease is yet unknown. 30 consecutive patients (9 male; age 25–77 years) with histologically confirmed Cushing’s disease underwent transsphenoidal adenomectomy between October 2018 and September 2019. All patients received perioperative pharmacological thromboprophylaxis. Whole leg compression ultrasound was performed within one week after surgery (median 2 days) to exclude leg vein thromboses (primary endpoint). Preoperative laboratory values including plasma cortisol and various coagulation parameters were evaluated as secondary outcome measures. A comparison was made between patients with and without thrombotic events (*p* value ≤ 0.05). A total of 2 out of 30 patients (6.7%; CI 0.8–24.1%) presented with asymptomatic perioperative deep vein thromboses of the lower legs. Thrombosis patients differed not significantly from their counterparts with respect to age, sex, and comorbidities, but preoperative morning plasma cortisol was significantly higher in patients with venous thromboses (421.0 ± 49.5 μg/l vs. 188.1 ± 78.2 μg/l; *p* = 0.01). Moreover, von Willebrand factor activity was markedly increased in one case (409.0%) compared to the mean value obtained from 28 patients without phlebothromboses (146.9 ± 60.7%; *p* < 0.01). Perioperative asymptomatic leg vein thrombosis can be found with the aid of compression ultrasound in a considerable proportion of patients undergoing transsphenoidal adenomectomy for Cushing’s disease despite the administration of pharmacological thromboprophylaxis.

## Introduction

Cushing’s disease (CD) is a rare endocrinological disorder of endogenous hypercortisolism caused by an adrenocorticotropic hormone (ACTH) secreting pituitary adenoma^1^. This condition comprises a large proportion of approximately 80% of newly diagnosed patients with Cushing’s syndrome (CS), which in turn is defined as systemic cortisol excess for any reason^2^. Prevalence studies across different countries suggest that approximately 1 per 1000 people of the general population harbor clinically and radiologically confirmed symptomatic pituitary adenomas with CD representing a fraction of about 4–6%^3^, whereas about 15% of surgically resected pituitary adenomas are functional ACTH staining adenomas^2,4^. This endocrinological condition comes along with increased morbidity resulting from metabolic, skeletal, infectious, and neurological/psychiatric complications, as well as mortality, mostly related to cardiovascular sequelae inclusive of coagulopathy^5^. Hypercoagulability and increased risk of thromboembolism has been determined in the majority of cohort studies on endogenous Cushing’s syndrome, whereby there appears to be a rapid and potentially reversible effect on clotting factor concentration (mainly coagulation factor VIII and von Willebrand factor) and a sustained impact on the vessel wall^6^. While the incidence of spontaneous symptomatic thromboembolic events is known to be significantly elevated in patients with CS compared with the general population (odds ratio of 17.8 in a recent systematic meta-analysis), there is a lack of information on asymptomatic venous thrombotic events of the lower extremity (VTE-L) in patients with CD^7^. We therefore aimed to systematically determine the prevalence of incidental perioperative VTE-L under prophylactic anticoagulation in a prospective consecutive series of 30 CD patients undergoing transsphenoidal surgery (TSS) by means of early postoperative whole leg compression ultrasound.

## Patients and methods

### Ethical standards and guideline compliance

The study protocol was approved by the ethics committee of the Hamburg Medical Council (registration number WF-020/19). Data collection and assessment are in line with the Declaration of Helsinki adopted by the 18th World Medical Association General Assembly in 1964 and its later amendments^8^. Where appropriate, data processing and presentation adheres to the strengthening the reporting of observational studies in epidemiology (STROBE) guidelines^9^**.**

### Study design and course

**Figure 1:**
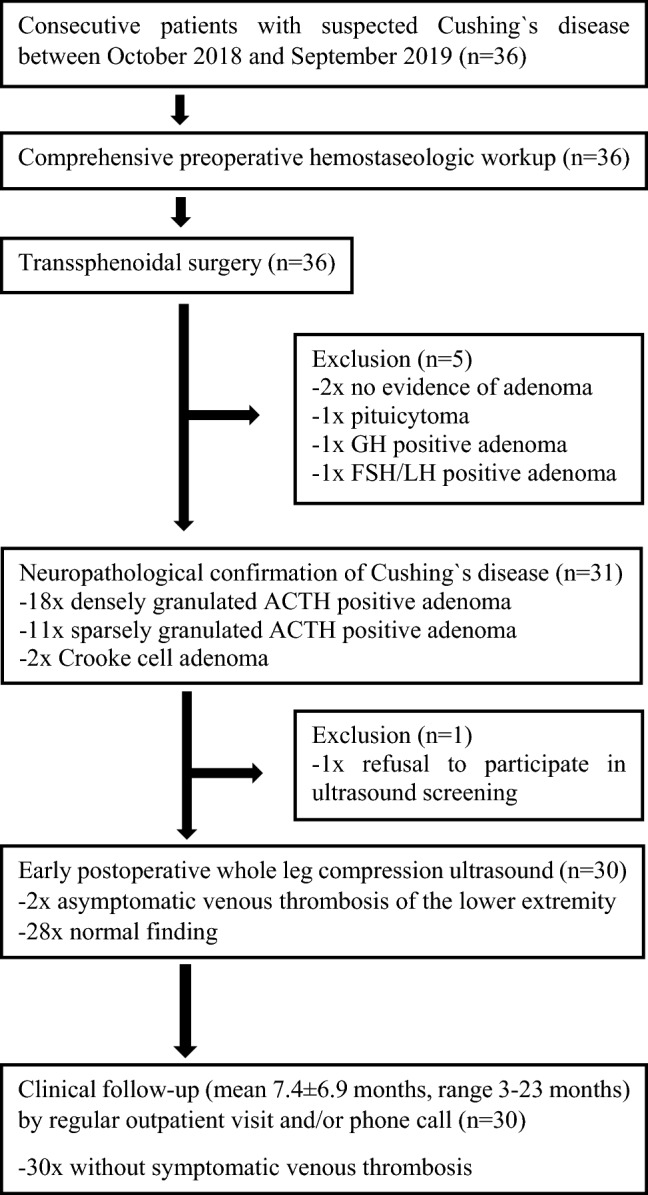
Study protocol. This flowchart outlines the study course along with the associated investigations and the reasons for excluding individual cases.

Due to the reported increased thromboembolic burden in CS, especially in the early postoperative setting^10^, all CD patients undergoing TSS at our tertiary pituitary referral center were offered an early postoperative VTE-L ultrasound screening during their hospital stay as of October 2018, in accordance with our routine institute’s internal treatment workflow. This study represents an analysis of asymptomatic perioperative VTE-L in all consecutive patients who underwent TSS for biochemically and neuropathologically confirmed CD at our pituitary surgery service between October 2018 and September 2019. Briefly, all patients with clinical/biochemical suspicion of CD during this period (n = 36) underwent TSS under total intravenous anesthesia by an experienced pituitary surgeon (J.F.). The diagnosis of CD was neuropathologically confirmed in 31 patients (n = 18 densely granulated ACTH positive adenoma, n = 11 sparsely granulated ACTH positive adenoma, n = 2 Crooke cell adenoma). The reasons for exclusion of the remaining five cases as well as the detailed study course can be found in Fig. 1. The 31 patients with a confirmed diagnosis of CD were offered early postoperative (< 1 week after surgery) compression ultrasound screening of both lower extremities by an experienced angiologist (G.A.) to either detect or rule out asymptomatic VTE-L (primary outcome measure). All but one patient agreed to undergo the screening (n = 30; 9 males; age 25–77 years). Ultrasonography scanning was performed using the Logiq E9 device from GE Healthcare (Chicago, Illinois, USA; www.gehealthcare.com). Cases with (n = 2) and without (n = 28) perioperative VTE-L detected by ultrasound were statistically compared with respect to demographic/clinical variables, various preoperative laboratory tests including comprehensive coagulation workup and endocrinological parameters as well as prior history of venous thrombotic events and/or pulmonary embolism as secondary outcome measures. In addition, all 30 CD patients were examined during regular follow-up (either outpatient appointment or telephone interview for patients living far away) with regard to the occurrence of symptomatic venous thromboembolic events in the longer-term postoperative course (mean follow-up 7.4 ± 6.9 months, range 3–23 months). Peri- and postoperative pharmacological thromboprophylaxis with low molecular weight heparin (40 mg enoxaparin sodium subcutaneously once daily) was used in all cases for at least 2 weeks after surgery. Patients were early mobilized postoperatively.

### Diagnostic criteria

#### Diagnosis of Cushing’s disease and early postoperative remission

CD was diagnosed based on the criteria outlined by Zada in 2013^2^. Shortly summarized, patients with clinical suspicion of CS were evaluated by at least two of the established hypercortisolism screening tests (i.e. 24-h urinary cortisol, midnight salivary cortisol, low-dose dexamethasone suppression test) to confirm the diagnosis of CS. The screening tests and their respective evidence are described in detail by Nieman et al.^11^ in a dedicated Endocrine Society clinical practice guideline. Subsequent serum ACTH level differentiated ACTH-dependent from ACTH-independent hypercortisolemia. In case of an ACTH-dependent CS, the presence of CD was verified by pituitary magnetic resonance imaging (supplemented, if necessary, by dynamic coronal contrast enhanced sequences to increase sensitivity and specificity^12^**)** and high-dose dexamethasone suppression test (+/− corticotropin-releasing hormone stimulation test). In doubtful cases cavernous sinus ACTH sampling was performed by our neuroradiological colleagues to identify an elevated central-peripheral ACTH ratio, which allows differentiation of CD from ectopic ACTH secretion, as well as an intercavernous gradient for the correct lateralization of the adenoma^13,14^. Early postoperative biochemical remission was defined as subnormal morning cortisol level (≤ 50 μg/l) on the first or second postoperative day without glucocorticoid replacement according to Esposito and colleagues^15^. For patients with a postoperative cortisol nadir below this threshold, a good long-term remission rate of almost 90% has been documented^16^.

#### Diagnosis of leg vein thrombosis

VTE-L is generally understood to be a partial or complete occlusion of a conducting or muscular vein of the lower extremity by a blood clot. With persistent procoagulant factors exceeding the fibrinolytic capacity and due to the slowing of blood flow, there is a risk of ascending appositional growth of the thrombus and eventually pulmonary embolism^17^. VTE-L was diagnosed using compression ultrasound of the entire lower extremity, which is the current imaging method of choice for confirmation and exclusion of this condition^17,18^. A recent large meta-analysis emphasizes the exceptionally high sensitivity (94.0%) and specificity (97.3%) of this diagnostic method in the detection of VTE-L^19^. The examination procedure followed the recommendations of Valentin et al.^20^ with one-stage sequential screening and documentation of the compressibility of the common, deep, and superficial femoral veins as well as the popliteal, peroneal, anterior/posterior tibial, and large muscular veins. Both legs were assessed in all patients.

### Patients

The patient cohort is composed of all consecutive adult patients who underwent TSS at our tertiary pituitary referral center between October 2018 and September 2019 for clinical, biochemical, and optionally magnetic resonance imaging suspicion of CD, whose diagnosis could be verified neuropathologically, and who were willing to undergo the routine ultrasound VTE-L screening implemented and recommended in our regular postoperative treatment algorithm. A total of 30 cases were collected. Among the 30 cases aged between 25 and 77 years, there were 9 men. Table 1 compares key demographic and clinical characteristics of CD patients with and without perioperative VTE-L, with no statistically significant difference observed for any variable in the group comparison.

**Table 1:** Demographic and clinical baseline characteristics of all included Cushing’s disease (CD) patients.

	CD with PVT (n = 2)	CD without PVT (n = 28)	*P* value
Age (years; median, range)	59 (40–77)	46 (25–65)	0.44*
Male (fraction, %)	2/2 (100.0)	7/28 (25.0)	0.08**
ASA physical status score (median, IQR)	2.5 (2.0–3.0)	2.0 (2.0–3.0)	> 0.99*
Coronary artery disease (fraction, %)	0/2 (0.0)	3/28 (10.7)	> 0.99**
Arterial hypertension (fraction, %)	1/2 (50.0)	17/28 (60.7)	> 0.99**
Obesity (fraction, %)	2/2 (100.0)	19/28 (67.9)	> 0.99**
Diabetes mellitus (fraction, %)	1/2 (50.0)	11/28 (39.3)	> 0.99**
Dyslipidemia (fraction, %)	0/2 (0.0)	3/28 (10.7)	> 0.99**
Limited mobility (fraction, %)	0/2 (0.0)	3/28 (10.7)	> 0.99**
Malignant tumor (fraction, %)	0/2 (0.0)	0/28 (0.0)	> 0.99**
Chronic renal failure (fraction, %)	1/2 (50.0)	2/28 (7.1)	0.19**
Smoking (fraction, %)	1/2 (50.0)	8/28 (28.6)	0.52**
Alcohol (fraction, %)	0/2 (0.0)	3/28 (10.7)	> 0.99**
Contraceptives (only females; fraction, %)	–	3/21 (14.3)	–
Anticoagulants/Antiplatelets (fraction, %)	0/2 (0.0)	3/28 (10.7)	> 0.99**
Coagulation disorder (fraction, %)	0/2 (0.0)	2/28 (7.1)	> 0.99**

*ASA* American society of anesthesiologists, *PVT* perioperative venous thrombosis of the lower extremity. *Mann–Whitney test. **Fisher’s exact test.

### Statistics

CD patients were separated into two groups depending on the ultrasound detection of perioperative VTE-L. Both cohorts were compared regarding demographic, clinical (comorbidities, cardiovascular risk factors, medications) and laboratory variables as well as with respect to the presence of symptomatic venous thromboembolic events in the history or in the further postoperative course. These data are presented descriptively by means of counts/percentages and central tendency measures supplemented by appropriate measures of dispersion (mean ± standard deviation (SD), median accompanied by interquartile range (IQR)), where suitable. For noncategorical variables, the Mann–Whitney test and the unpaired t test were used, and for categorical variables, the Fisher’s exact test was employed (two-tailed p value for all statistical test procedures). The level of significance was set at 0.05. Calculations were conducted with GraphPad Prism version 8.3.1 (GraphPad Software, San Diego, California, USA/www.graphpad.com).

### Conference presentation

Parts of this study were presented at the 64th annual meeting of the Society of Thrombosis and Haemostasis Research, which was held in Bremen, Germany, between February 18 and 21, 2020.

### Ethics approval

This study was performed in line with the principles of the 1964 Declaration of Helsinki and its later amendments. Approval was granted by the ethics committee of the Hamburg Medical Council (registration number WF-020/19).

### Consent to participate

Informed consent was obtained from all individual participants included in the study.

## Results

### Surgical outcome and early postoperative adverse events

Of the 30 consecutive adult patients (9 males; age range 25–77 years; median 46 years; IQR 38–54 years) with histopathologically confirmed CD following TSS at our university hospital between October 2018 and September 2019, 27 (90.0%) had a postoperative morning serum cortisol level below 50 myg/l under steroid withdrawal as an early sign of biochemical remission (preoperative/postoperative mean morning serum cortisol 203.6 ± 96.3 myg/l/27.6 ± 31.0 myg/l). Besides 2 ultrasonographically detected asymptomatic VTE-L in different CD patients with early remission after macroadenoma resection, 8 of 30 (26.7%) patients showed transient adverse events during the early postoperative course (n = 2 urinary tract infection, n = 3 diabetes insipidus, n = 1 pathologic rib fracture, n = 1 peritonsillar abscess, n = 1 thrombophlebitis of the wrist). All complications except for the peritonsillar abscess, which required surgical treatment, could be managed conservatively.

### Prevalence of perioperative asymptomatic leg vein thrombosis in Cushing’s disease

Early postoperative compression ultrasound screening (range 1.0–7.0 days; median 2.0 days, IQR 1.0–4.0 days) of both lower extremities of all 30 CD patients detected asymptomatic VTE-L in 2 cases (6.7%; 95% confidence interval 0.8–24.1%). One patient was diagnosed with thrombosis in the posterior tibial vein and in another case the muscular veins of the calf were affected.

### Demographic and clinical comparison between Cushing’s disease patients with and without perioperative asymptomatic leg vein thrombosis

No statistically significant differences were observed between CD patients with and without VTE-L with respect to demographic variables, physical condition, comorbidities, cardiovascular risk factors, and anticoagulant medication, as shown in Table 1. However, it is notable that both CD patients with VTE-L were male, whereas the proportion of males in the non-VTE-L group was only 7/28 (25.0%; *p* = 0.08).

### Laboratory value characteristics in Cushing’s disease patients with and without perioperative incidental deep vein thrombosis of the lower extremity

CD patients with perioperative VTE-L had significantly higher preoperative morning serum cortisol levels compared to their non-VTE-L counterparts (mean 421.0 ± 49.5 µg/l vs. 188.1 ± 78.2 µg/l; *p* = 0.01). Postoperatively, there was no group difference in cortisol measurements. There were also significant hemostaseological discrepancies in both cohorts with comparatively higher von Willebrand factor activity in case of perioperative VTE-L (mean 409.0 ± 0.0% vs. 146.9 ± 60.7%; *p* < 0.01) and lower fibrinogen level leading to prolonged thrombin time. Remarkably, the coagulation factor VIII was well above the reference value regardless of the occurrence of perioperative VTE-L, with no significant group difference observed. The two CD patients with VTE-L, both men, showed decreased testosterone levels (0.6 ± 0.1 µg/l; reference range 2.5–8.2 µg/l). The detailed laboratory results of both groups can be found in Table 2.

**Table 2:** Laboratory findings in Cushing’s disease (CD) patients with or without perioperative venous thromboses of the lower extremity (PVT).

	CD with PVT (n = 2)	CD without PVT (n = 28)	*P* value
Preoperative morning cortisol (mean, SD)	421.0 ± 49.5	188.1 ± 78.2	0.01**
Postoperative morning cortisol (mean, SD)	49.5 ± 14.9	26.0 ± 31.4	0.06**
Thrombocytes (mean, SD)	186.5 ± 89.8	314.4 ± 87.8	0.06**
C-reactive protein (mean, SD)	5.0 ± 0.0	11.4 ± 24.3	0.89**
Leukocytes (mean, SD)	10.5 ± 1.8	9.8 ± 2.6	0.47**
Hemoglobin (mean, SD)	14.8 ± 1.2	14.1 ± 1.9	0.78**
Hematocrit (mean, SD)	43.2 ± 1.8	42.8 ± 5.5	0.96**
Creatinine (mean, SD)	1.3 ± 0.2	1.0 ± 0.2	0.07**
International Normalized Ratio (mean, SD)	1.0 ± 0.1	0.9 ± 0.1	0.65**
Activated partial thromboplastin time (mean, SD)	23.5 ± 0.7	26.0 ± 2.5	0.16**
Thrombin time (mean, SD)	17.5 ± 1.6	13.3 ± 0.8	0.01**
Fibrinogen (mean, SD)	1.8 ± 0.2	3.5 ± 1.3	0.01**
D-dimer (mean, SD)	1.0 ± 0.0*	0.8 ± 1.5	0.89***
Coagulation factor VIII (mean, SD)	253.5 ± 0.0*	191.7 ± 79.4	0.45***
Von Willebrand factor activity (mean, SD)	409.0 ± 0.0*	146.9 ± 60.7	< 0.01***

All values except the postoperative cortisol were obtained preoperatively. Reference range for laboratory results: morning serum cortisol 63.0–181.0 µg/l; thrombocytes 150.0–400.0 billions/l; C-reactive protein 0.0–5.0 mg/l; leukocytes 3.8–11.0 billions/l; hemoglobin 12.3–15.3 g/dl; hematocrit 35.0–45.0%; creatinine 0.5–1.0 mg/dl; International Normalized Ratio −; activated partial thromboplastin time 25.0–36.0 s; thrombin time 10.3–16.6 s; fibrinogen 2.0–3.9 g/l; D-dimer < 0.5 mg/l; coagulation factor VIII 50.0–150.0%; von Willebrand factor activity 48.8–163.4%. SD standard deviation. *Only one value available. **Mann–Whitney test (two-sided *p* value). ***Unpaired t test (two-sided *p* value). Significant values are in [bold].

### Symptomatic thromboembolic events during the course of Cushing’s disease

A total of 2 out of 30 (6.7%) CD patients in our case series had a history of previous clinically manifest thromboembolic events (n = 2 prior VTE-L without subsequent progression to pulmonary embolism). No symptomatic venous thromboembolism occurred during the clinical follow-up of 7.4 ± 6.9 months (range 3–23 months, none of the patients lost to follow-up) under postoperative prophylactic anticoagulation with low molecular weight heparin, which was administered subcutaneously to all patients for at least 2 weeks after surgery. In addition, the two patients with sonographically detected asymptomatic perioperative VTE-L received weight-adapted therapeutic anticoagulation (1 mg enoxaparin sodium/kilogram body weight subcutaneously 2 times per day) from the time of diagnosis for a period of 3 months. There was no significant difference in the incidence of symptomatic venous thromboembolism in the overall disease course in either group, as outlined in Table 3.

**Table 3:** Occurrence of symptomatic venous thrombotic events (VTE) before and after transsphenoidal surgery for Cushing’s disease (CD) in patients with and without asymptomatic perioperative venous thrombosis (PVT) of the lower extremity under anticoagulant protection with low molecular weight heparin (LMWH).

	CD with PVT (n = 2)	CD without PVT (n = 28)	*P* value
Preoperative history of symptomatic VTE (fraction, %)	1/2 (50.0)	1/28 (3.6)	0.13*
Postoperative symptomatic VTE** (fraction, %)	0/2 (0.0)	0/28 (0.0)	> 0.99*
Postoperative anticoagulation with LMWH ≥ 2 weeks (fraction, %)	2/2 (100.0)	28/28 (100.0)	> 0.99*

*Fisher’s exact test (two-sided). **Mean clinical follow-up 7.4 ± 6.9 months, minimum 3 months.

## Discussion

We present here the first attempt to determine the prevalence of asymptomatic perioperative VTE-L in a consecutive series of CD patients undergoing adenomectomy. The surgical outcome (90% early biochemical remission) as well as the described perioperative complication rate are comparable to published series of experienced pituitary surgery centers^2,21,22^, which is relevant for the generalizability of the results. We found incidental VTE-L in approximately 7% of CD patients by early postinterventional compression ultrasound of the lower extremities following TSS. While large meta-analyses have already demonstrated that patients with endogenous CS have a significantly increased risk of spontaneous thromboembolic events compared to the general population (odds ratio 17.8) and the incidence of early postoperative symptomatic venous thromboembolic events ranges between 1.2 and 4.75%, data on the hazard of clinically silent perioperative VTE-L in CD patients undergoing pituitary surgery have been lacking so far^7,22^. Asymptomatic distal VTE-L may progress through appositional growth in the presence of persistent propagation factors (i.e. increased clotting activity and/or inhibited fibrinolysis) and ultimately lead to symptomatic proximal deep vein thromboses or potentially fatal pulmonary embolism^17,23,24^. The clinical relevance of asymptomatic deep vein thrombosis was described in a large collective of patients hospitalized for acute medical conditions. In the presence of sonographically detected asymptomatic venous thrombosis, short-term all-cause mortality was increased almost threefold compared with the control group without thrombotic events^25^. This is of great importance because thromboembolism, along with other cardiovascular events and serious infections including sepsis, are major contributors to the high mortality burden in CD and it has been repeatedly and consistently reported that the risk of thrombosis is especially high in the postoperative period up to 3 months^10,26–28^. Patients with and without incidental perioperative VTE-L showed no significant differences with regard to demographic and clinical parameters. However, the small number of VTE-L patients in our series (n = 2) compromises the statistical analysis of these parameters, so the results here should be viewed with caution. In a larger CD patient population, which is difficult to reach because of the rarity of this condition, it may be possible to identify demographic and clinical risk factors in the future. Interestingly, a nonsignificant trend toward male sex was identifiable in the presence of VTE-L. Consistent with our results, Suarez et al. also found no relevant dependence of thrombosis risk in CS patients with respect to demographic variables, cardiovascular risk factors, and comorbidities^10^. A proportion of almost 7% of the CD patients in our consecutive series reported a symptomatic thromboembolism in their preoperative medical history, whereas no such event occurred in any of these patients during the postoperative follow-up period, which averaged more than 7 months, when they were receiving either prophylactic anticoagulation for at least 2 weeks after TSS or, in the case of detected asymptomatic VTE-L, longer-term weight-adjusted therapeutic anticoagulation with a low molecular weight heparin. The analogous treatment of incidental and symptomatic VTE-L is suggested in the German guideline^17^, whereas the American guideline recommends this approach in patients without severe symptoms only in the presence of certain risk factors, such as a nonreversible provoking factor, inpatient status, or active cancer^29^. Comparing the relative risk of venous thrombotic events in Cushing’s patients (versus the normal population) with that of cancer patients in general, the risk of a thromboembolic event is markedly higher in Cushing's syndrome^7,30^. Therefore, therapeutic anticoagulation of the two CD patients with VTE-L seems justified according to both guidelines mentioned above. While the stated frequency of preoperative symptomatic thromboembolic events is well in line with the literature^10,28^, our results regarding the incidence of these events in the postoperative course after TSS are at the lower limit of the 0–6% spectrum reported in the review article by van der Pas^27^. This encouraging low rate is probably related to early mobilization and the consistent use of anticoagulant prophylaxis/therapy in all investigated CD cases, which has been shown to significantly reduce the risk of postoperative thromboembolic events in this specific patient population^31^. In addition, early postoperative ultrasound screening may have detected asymptomatic distal VTE-L in CD patients at an early stage, which, if left untreated, might have later developed into symptomatic proximal VTE-L. To date, consensus-based recommendations for peri- and postoperative thromboprophylaxis in patients with CD are lacking, and various drug regimens have been proposed in the past, including weight-adjusted low molecular weight heparin for one month or even prophylactic anticoagulation for up to 60 days after pituitary surgery^10,32^. The extended 2-month period is supported by a multicenter cohort study in which the majority of postoperative thrombotic events in patients with ACTH-dependent CS were diagnosed within 2 months of surgery, at which time, interestingly, thromboprophylaxis was no longer administered in most of these patients^28^. To better assess the probability of thrombotic events in patients with CS, a disease-specific risk score was developed several years ago, which may provide guidance concerning the pre- and postoperative necessity and duration of medical thromboprophylaxis in CD patients^33^. In general, for initial thrombosis therapy, it has been shown that a once-daily low molecular weight heparin application is not disadvantageous compared with a twice-daily drug regimen^34^. The diagnosed asymptomatic VTE-L in our study were all detected in the distal lower extremity (posterior tibial vein, calf muscle vein). For these so-called isolated distal vein thromboses, a prognostically more favorable course with less frequent recurrences and lower rates of postthrombotic syndrome has been described compared with proximal thrombotic events. In general, isolated distal vein thrombosis is treated analogously to proximal deep vein thrombosis by therapeutic anticoagulation for a period of up to 3 months, although this recommendation is currently not based on high-level evidence and it is unclear whether prolonged or shortened anticoagulation would be beneficial for certain patient groups^17^. Given the unclear evidence regarding the optimal treatment of isolated distal VTE-L in general and particularly in the context of CD, we recommended an early outpatient consultation with a vascular specialist for the affected patients. A recently published Cochrane Review found also high-quality evidence for the effectiveness of graduated compression stockings regarding mechanical VTE-L prevention in hospitalized patients undergoing general and orthopedic surgery, but the transferability of these results to pituitary surgery patients is speculative at this time^35^. We found an increase in mean preoperative morning cortisol levels as well as von Willebrand factor activity and lower fibrinogen levels in CD patients with perioperative asymptomatic VTE-L compared to their counterparts without such events. Most studies have shown that the hypercoagulable state in endogenous CS is caused by elevated levels of procoagulant factors, mainly coagulation factor VIII and von Willebrand factor, and also by impaired fibrinolytic capacity (elevated plasminogen activator inhibitor 1, among others)^7,36^. Von Willebrand factor has a dual role in hemostasis: on the one hand it promotes platelet adhesion by anchoring them to the subendothelial matrix of damaged vessels, on the other hand it is a carrier protein that protects coagulation factor VIII from proteolytic degradation^37^. Elevated coagulation factor VIII and von Willebrand factor levels are known to be associated with an increased risk of thrombosis^38^. Regarding fibrinogen concentration alterations, controversial results have been reported for CS in different studies^6,27^. Following CS remission, there is usually an improvement in the previously profound coagulatory abnormalities^39,40^. Interestingly, D-dimer levels, as a well-recognized marker of thrombosis, were equally moderately elevated in both CD patient groups regardless of the presence of asymptomatic VTE-L. Elevated D-dimer levels in CD patients compared with the general population have been consistently shown in previous studies^36,41^. The undetectable influence of asymptomatic VTE-L on D-dimer levels in CD patients in our study may be related to the fact that D-dimer assays have a significantly poorer performance even for symptomatic distal deep vein thromboses compared with proximal thrombotic events^42^. Thus, it may be expected that asymptomatic (i.e., usually less severe) distal VTE-L will have even less effect on D-dimer levels. Furthermore, as already discussed, the limitations of the statistical analysis resulting from the small number of VTE-L patients should be considered.

### Strengths and limitations

The major limitation of the investigation is the limited generalizability of the results due to the study design, as it is a monocentric case series. This is related to the moderate sample size of 30 patients. Another shortcoming is the small number of asymptomatic VTE-L (n = 2), which substantially restricts statistical comparison between patients with and without thrombotic events and prevents detection of potential predictors of VTE-L. Moreover, it should be noted that because ultrasound screening for the detection of asymptomatic VTE-L was performed only once in the early postoperative setting in our study, no definitive statement can be made regarding the exact time of occurrence of these thrombotic events in the perioperative period. We therefore deliberately use the term prevalence instead of incidence here. However, screening was purposely carried out at this time because multiple prothrombotic risk factors accumulate here (endocrine predisposing disorder, postoperative immobility, recent surgical trauma) and thus the highest risk of thrombosis is to be expected here. On the other hand, the quality of the study is significantly enhanced because the selection bias could be eliminated by the fact that, apart from one individual who refused to participate, all consecutive CD patients of the study period could be screened for VTE-L by experienced angiologists using compression ultrasound. In addition, included patients had an average follow-up of more than 7 months (minimum 3 months), so that a sufficient statement about the further clinical course can be made.

## Conclusions

Now that it has been shown that clinically silent perioperative VTE-L is not uncommon in CD despite pharmacological thromboprophylaxis, further large-scale compression ultrasound screening studies are needed to determine the risk of progression to symptomatic venous thromboembolic events in these patients. With the help of this important clinical information and by including further parameters such as the comprehensive coagulation status and the above-mentioned thrombosis risk score developed specifically for CS patients by Zilio et al.^33^, it may be possible in the future to better implement medical thromboprophylaxis tailored to the individual risk of the respective CD patient. However, as long as the benefit of ultrasound thrombosis screening has not been proven in prospective clinical studies, no general recommendation can be made in this regard.

## Data Availability

All data generated or analyzed during this study are included in this published article.

## Abbreviations

ACTH: Adrenocorticotropic hormone
CD: Cushing’s disease
CS: Cushing’s syndrome
IQR: Interquartile range
SD: Standard deviation
TSS: Transsphenoidal surgery
VTE-L: Venous thrombotic event(s) of the lower extremity
